# Clinical Characteristics and Diagnostic Pitfalls of Pheochromocytoma: A Pathology-Confirmed Retrospective Study From a Single Japanese Center

**DOI:** 10.7759/cureus.108629

**Published:** 2026-05-11

**Authors:** Takashi Sekido, Shohei Takayama, Yusuke Shibata, Hiroyuki Sagesaka, Yasuho Shimada, Satoshi Kubota, Takayuki Karasawa, Yuko Kawai, Masanori Yamazaki, Mitsuhisa Komatsu

**Affiliations:** 1 Diabetes and Endocrinology, Shinshu University Hospital, Matsumoto, JPN; 2 Diabetes, Endocrinology, and Metabolism, Shinshu University School of Medicine, Matsumoto, JPN

**Keywords:** 123i-metaiodobenzylguanidine scintigraphy, enhanced-computed tomography, extra-adrenal paraganglioma, magnetic resonance image, pheochromocytoma

## Abstract

Objective: Pheochromocytoma poses diagnostic challenges, especially in small or atypical tumors. Despite the use of various imaging modalities, their preoperative diagnostic accuracy remains limited. The study aimed to assess pathologically confirmed cases of pheochromocytoma and clarify diagnostic pitfalls and various imaging findings.

Patients and methods: We retrospectively analyzed 47 pathologically confirmed pheochromocytomas treated at our institution between 2015 and 2022. Clinical characteristics, biochemical results, and imaging findings [computed tomography (CT), magnetic resonance imaging (MRI), and ^123^I-metaiodobenzylguanidine (MIBG) scintigraphy] were reviewed. MRI scans were independently reviewed by two board-certified radiologists, with consensus on discrepancies.

Results: The mean patient age was 59.3 years, and the mean tumor size was 4.26 cm. The ^123^I-MIBG scintigraphy positivity rate was 85.1%. Characteristic MRI features, including high signal intensity on T2-weighted images, internal heterogeneity, early enhancement, and lack of signal drop on fat-suppressed T2 images, were observed in >90% of cases. No adverse events occurred among 31 patients who underwent contrast-enhanced CT without α-blocker pretreatment. Three tumors were non-functional and ^123^I-MIBG-negative, diagnosed only via postoperative pathology.

Conclusion: Diagnosing pheochromocytoma remains challenging in small or atypical cases, emphasizing the value of combining multiple imaging modalities, particularly MRI. These findings highlight the importance of a multimodal approach in clinical practice.

## Introduction

Pheochromocytomas and paragangliomas are rare neuroendocrine tumors originating from chromaffin cells in the adrenal medulla or extra-adrenal paraganglia. Excessive catecholamine secretion can cause life-threatening cardiovascular complications. Common symptoms include hypertension, palpitations, headaches, and diaphoresis; however, some patients remain asymptomatic and are detected incidentally [1].

Diagnosis is based on biochemical confirmation of catecholamine overproduction, typically via plasma or urinary metanephrines. Tumor localization depends on imaging modalities such as computed tomography (CT) and magnetic resonance imaging (MRI); functional imaging, such as ^123^I-metaiodobenzylguanidine (MIBG) scintigraphy, is reserved for complex cases [2,3]. Surgery remains the definitive treatment, necessitating preoperative alpha-blockade and fluid resuscitation to prevent hypertensive crises [4].

In Japan, specific challenges include risks of hypertensive crises associated with contrast-enhanced CT, preference for MRI owing to safety concerns, and inconsistencies in preoperative optimization [5,6]. This study reviewed pheochromocytoma cases diagnosed and managed at our institution over seven years. The study aimed to retrospectively assess pathologically confirmed cases of pheochromocytoma at our institution to clarify diagnostic pitfalls and various imaging findings. We hypothesized that a multimodal imaging approach is necessary to improve preoperative diagnostic accuracy, particularly in atypical cases.

## Materials and methods

We retrospectively reviewed 47 pathologically confirmed cases of pheochromocytoma treated at Shinshu University Hospital between April 1, 2015, and March 31, 2022. Clinical and biochemical data were extracted from medical records. Biochemical data included serum catecholamines (CA test, Tosoh Corporation, Tokyo, Japan), urine catecholamines (CA test, Tosoh Corporation), serum metanephrines (2-MET Plasma ELISA kit, DENIS Pharmacia, Tokyo, Japan; measured using an Infinite F50R microplate reader, Tecan Japan, Kawasaki, Japan), and urine metanephrine levels (in-house reagent, LSI Medience Corporation, Tokyo, Japan). Abnormal values were defined as above normal for serum metanephrine and more than three times the upper limit for urine catecholamine and urine metanephrine, based on the Japan Endocrine Society clinical practice guideline [7]. Imaging data included tumor size and findings from computed tomography (CT; Revolution HD or Revolution CT, GE Medical Systems, Milwaukee, USA), magnetic resonance imaging (MRI; MAGNETOM Vida, MAGNETOM Prisma, Siemens Healthineers, Munich, Germany), and functional imaging such as ^123^I-metaiodobenzylguanidine (MIBG) scintigraphy (Symbia T6, Siemens Healthineers, Munich, Germany). Imaging findings from CT, MRI, and ^123^I-MIBG scintigraphy were independently reviewed by two board-certified radiologists blinded to biochemical data. Discrepancies were resolved via consensus.

We also evaluated cases with negative MIBG scintigraphy findings and those who underwent contrast-enhanced CT without alpha-blocker pretreatment.

Imaging evaluation

CT examinations were performed using multidetector scanners with or without intravenous contrast enhancement based on clinical indication. MRI was conducted using standard adrenal imaging protocols, including T1-weighted, T2-weighted, in-phase, and opposed-phase T1-weighted images. Early enhancement patterns were evaluated on dynamic contrast-enhanced imaging where available.

^123^I-metaiodobenzylguanidine (MIBG) scintigraphy was interpreted by experienced nuclear medicine physicians, and tracer uptake in the adrenal lesion exceeding background physiological uptake was considered positive.

MRI findings were evaluated according to previously reported radiological characteristics of pheochromocytoma, including high signal intensity on T2-weighted imaging, internal heterogeneity, early contrast enhancement, and fat suppression on in-phase and opposed-phase T1-weighted images.

The imaging features used in this study were derived from previously published radiological characteristics of pheochromocytoma and do not involve any proprietary scoring system or licensed diagnostic tool.

Statistical analysis was performed using the Mann-Whitney U test, and a p-value <0.05 was considered statistically significant. This study was approved by the Ethics Committee of the Shinshu University School of Medicine (approval no. 3959). This study was conducted using an opt-out consent approach.

## Results

The clinical characteristics of the patients are summarized in Table 1.

**Table 1:** Patient characteristics. ^a^ + means accumulation on ^123^I-metaiodobenzylguanidine (MIBG) scintigraphy. ^b^ + means high signals on T2-weighted MR images. ^c^ + means tumor parenchyma shows heterogeneous signals on MR images. ^d^ + means lack of fat suppression on in-phase and opposed-phase T1-weighted images. ^e^ − means no attempt at enhancement, + means the cases with enhanced CT. ^f^ + means over the normal serum metanephrine level (0−130 pg/mL),  normal serum normetanephrine level (0−506 pg/mL), more than three times the normal urinary metanephrine level (0.05−0.20 mg/d), or more than three times the normal urinary normetanephrine level (0.10−0.28 mg/d).

Case number	Age	Sex	Tumor size (cm) maximum diameter	Scintigraphy^a^	MRI T2 high signal^b^	MRI heterogeneous^c^	MRI early enhancement	MRI fat supression^d^	Enhanced CT^e^	HU value in enhanced CT	MT or NMT high^f^
1	65	F	3.5	＋	+	+	+	NA	−	NA	−
2	52	M	6.6	＋	+	+	NA	NA	−	NA	+
3	74	M	3.8	＋	NA	NA	NA	NA	+	124.39	+
4	69	F	7.3	＋	+	+	+	−	+	76.62	+
5	41	F	5.2	＋	+	+	+	−	+	155.37	+
6	55	F	2.8	＋	+	+	+	−	+	86.7	+
7	67	F	3	＋	+	+	+	−	+	117.1	+
8	64	M	5.1	＋	NA	NA	NA	NA	+	81.02	+
9	34	M	3.8	＋	+	+	NA	−	−	NA	+
10	48	F	2.4	＋	+	+	NA	−	−	NA	−
11	59	F	3	＋	+	+	+	−	−	NA	−
12	49	F	4.7	＋	+	+	+	−	+	146.69	+
13	40	M	2	＋	NA	NA	NA	NA	+	107.56	+
14	40	M	2.2	＋	+	+	NA	−	+	82.95	+
15	56	M	1.7	＋	+	-	NA	−	+	83.41	NA
16	55	F	2.3	＋	+	-	NA	−	+	129.9	+
17	77	M	2.3	＋	+	+	+	−	+	84.59	+
18	60	M	4.9	＋	+	+	NA	−	+	210.05	+
19	44	F	2.8	＋	+	+	+	−	−	NA	+
20	73	M	4.9	＋	+	+	+	−	+	115.22	+
21	65	F	4.4	＋	+	+	+	−	−	NA	+
22	78	F	2.8	＋	+	+	+	−	−	NA	+
23	63	F	4.3	＋	+	+	+	+	+	52.38	−
24	43	F	6	＋	+	+	+	−	+	93.6	+
25	69	F	1.2	＋	+	+	+	−	+	125.39	−
26	33	F	2.5	−	+	+	NA	−	+	218.39	+
27	40	F	1.4	＋	+	+	−	−	+	130.23	−
28	59	F	5.5	＋	+	+	+	−	−	NA	+
29	56	F	2	＋	+	+	NA	NA	−	NA	+
30	16	M	4.1	＋	+	+	+	−	+	109.98	−
31	67	F	1.1	−	+	+	NA	−	+	107.07	−
32	71	F	2.8	＋	+	+	NA	−	−	NA	+
33	78	M	1.5	＋	+	+	NA	+	+	39.41	−
34	76	M	32	＋	+	+	NA	NA	−	NA	+
35	64	M	4.3	−	+	+	NA	−	+	103.61	+
36	86	M	3.6	＋	+	+	NA	−	+	125.71	NA
37	71	F	4.2	＋	+	+	NA	−	−	NA	+
38	49	F	8	−	+	+	NA	−	+	82.11	−
39	70	M	3.3	＋	+	+	NA	−	+	120.63	+
40	60	F	10	＋	+	+	+	−	+	60.57	+
41	67	M	2.2	＋	−	−	+	−	+	123.44	+
42	54	F	4	＋	+	+	NA	−	+	100.23	+
43	57	M	7	＋	+	+	+	−	−	NA	+
44	57	F	2.8	−	+	+	NA	−	+	120.66	+
45	68	M	2.5	−	+	+	NA	NA	−	NA	NA
46	71	F	1.8	＋	+	+	NA	−	−	NA	+
47	79	M	0.9	−	+	+	+	−	+	92.95	−
Mean	59		4.3							109.93	
SD	14		4.6							38.28	

It includes age, sex, tumor size, and imaging findings from scintigraphy, MRI, and CT. The mean age was 59.3 ± 14.4 years, with 57.4% female. The average tumor diameter was 4.26 ± 4.56 cm. MIBG scintigraphy was positive in 40/47 cases (85.1%). The Mann-Whitney U test showed no significant differences between MIBG-positive and MIBG-negative groups in age (U = 134.5, p = 0.963) or tumor diameter (U = 177.5, p = 0.269).

We evaluated four key MRI features considered essential for diagnosing pheochromocytomas: high T2-weighted signal intensity, internal heterogeneity, early contrast enhancement, and absence of high signal intensity on fat-suppressed T2-weighted images. These features were present in 97.7%, 93.2%, 95.4%, and 94.5% of cases, respectively, indicating their strong diagnostic marker potential.

**Figure 1:**
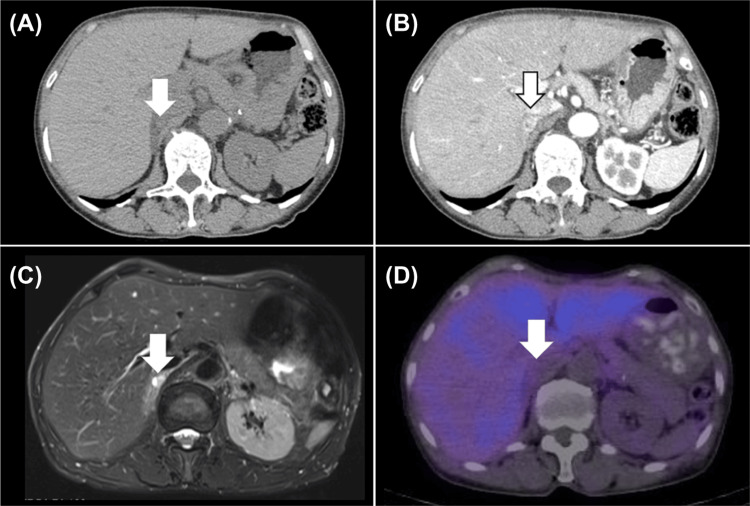
Imaging examination of the right adrenal tumor in case 31. (A) Plain CT. (B) Contrast-enhanced CT. (C) T2-weighted MRI. (D) 123I-MIBG scintigraphy. (A) Unenhanced CT image showing a small right adrenal mass. (B) Contrast-enhanced CT demonstrating mild enhancement of the lesion. (C) T2-weighted MRI revealing high signal intensity within the tumor. (D) ¹²³I-MIBG scintigraphy showing negative uptake in the right adrenal gland. ^123^I-MIBG: ^123^I-metaiodobenzylguanidine.

**Figure 2:**
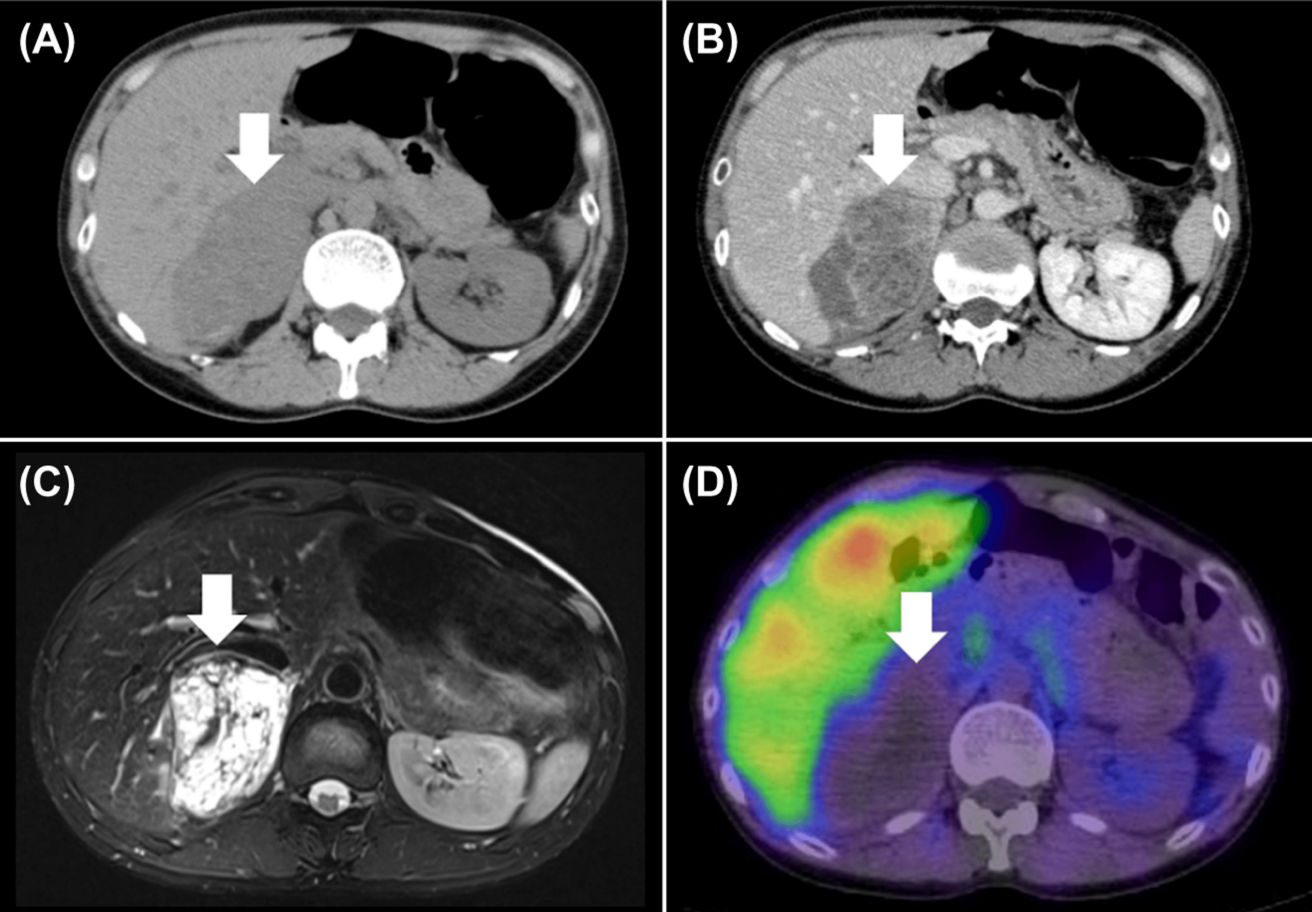
Imaging examination of the right adrenal tumor in case 38. (A) Plain CT. (B) Contrast-enhanced CT. (C) T2-weighted MRI. (D) 123I-MIBG scintigraphy. (A) Unenhanced CT image showing a right adrenal mass. (B) Contrast-enhanced CT with moderate enhancement of the tumor. (C) T2-weighted MRI showing marked hyperintensity. (D) ¹²³I-MIBG scintigraphy showing negative uptake in the right adrenal gland. ^123^I-MIBG: ^123^I-metaiodobenzylguanidine.

**Figure 3:**
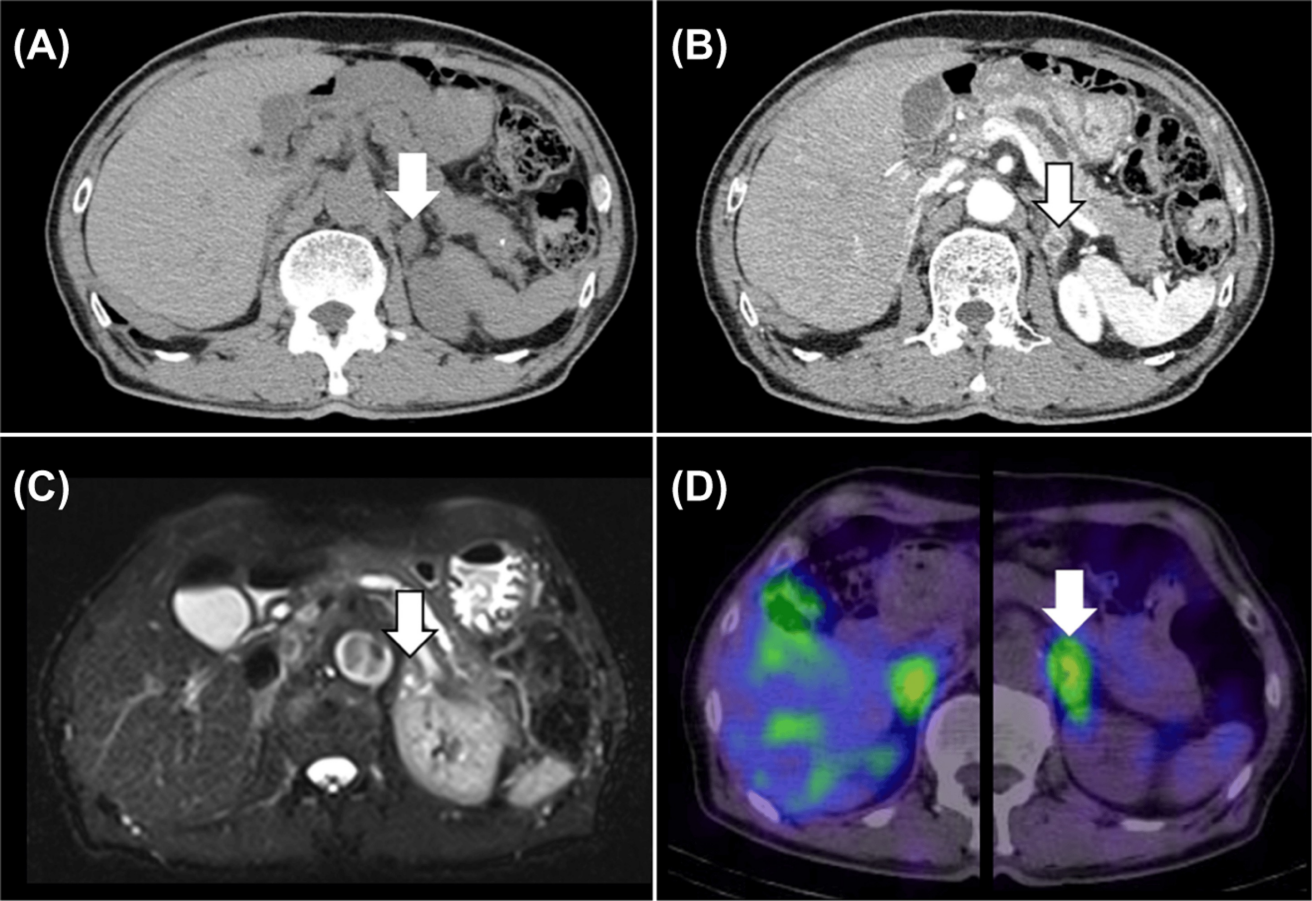
Imaging examination of the right adrenal tumor in case 47. (A) Plain CT. (B) Contrast-enhanced CT. (C) T2-weighted MRI. (D) 123I-MIBG scintigraphy. (A) Unenhanced CT image showing a small left adrenal lesion. (B) Contrast-enhanced CT demonstrating faint enhancement. (C) T2-weighted MRI exhibiting heterogeneous high signal intensity. (D) ¹²³I-MIBG scintigraphy demonstrated no laterality of uptake between the adrenal tumor and the contralateral adrenal gland, suggesting only physiological adrenal uptake. Therefore, the scan was considered negative for pheochromocytoma. ^123^I-MIBG : ^123^I-metaiodobenzylguanidine.

Contrast-enhanced CT was performed before diagnosis in 31/47 cases (66.0%), with no hypertensive crises reported. The average Hounsfield unit (HU) value in enhanced CT was 109.93±38.28. Elevated plasma normetanephrine or metanephrine levels were detected in 27 cases (57%), and elevated urinary levels in 33 cases (77%). Three patients (Cases 31, 38, and 47) were biochemically silent and ^123^I-MIBG-negative, with diagnosis confirmed only by postoperative pathology (Figures 1-3).

The mean age was 65.0 ± 15.1 years in the three biochemically silent cases and 58.9 ± 14.4 years in the remaining cases. The average tumor diameter was 3.3 ± 4.0 cm in the three cases and 4.3 ± 4.6 cm in the other 44 cases. Regarding the four key MRI features, high T2-weighted signal intensity, internal heterogeneity, early contrast enhancement, and absence of high signal intensity on fat-suppressed T2-weighted images, the three cases showed 100%, 100%, 100%, and 0%, respectively. The corresponding rates in the remaining 44 cases were 90.9%, 97.6%, 92.7%, and 95.7%, respectively. As only three cases were biochemically silent and ^123^I-MIBG-negative, statistical analysis was not feasible. No apparent differences were observed between the two groups.

## Discussion

This study summarizes the key findings from seven years of pheochromocytoma cases at our institution. Contrast-enhanced CT was conducted without prior alpha-blockade in 65.9% of cases, contrary to Japanese guidelines recommending pharmacological preparation with agents such as phentolamine to prevent hypertensive crises [7]. Although no adverse events occurred, this practice raised patient safety concerns. Nonionic contrast agents are generally considered safe [8]; however, crises following their use have been documented recently [5,6]. These findings highlight the importance of adhering to established protocols, particularly in settings with limited emergency response capacity.

Three patients were biochemically silent, showing negative MIBG scintigraphy and normal or marginally elevated plasma or urinary catecholamine levels. Unlike previous studies focusing on hypertensive crises [5,6], this study addressed diagnostic challenging cases. These studies highlight the challenges of identifying atypical tumors and support the use of multimodal strategies that incorporate advanced imaging and novel biomarkers [9-11]. Diagnostic evaluation of pheochromocytoma should integrate imaging and clinical context, especially when catecholamine assays and scintigraphy yield inconclusive results.

MRI consistently demonstrates high signal intensity on T2-weighted images, tumor heterogeneity, early contrast enhancement, and absence of signals on fat-suppressed T2-weighted images. Although T2 hyperintensity is sensitive, it lacks specificity, as other adrenal lesions may appear similar. Chemical shift imaging, diffusion-weighted imaging, and ^68^Ga-dodecanetetraacetic acid (DOTA) positron emission tomography (PET) may improve diagnostic accuracy, especially in biochemically silent cases [12-16]. MRI remains a valuable diagnostic tool, particularly when biochemical abnormalities are absent [17,18]. Alpha-blockade should be considered in suspected cases, even in the presence of negative laboratory and scintigraphic results [9]. Newer modalities such as ^68^Ga-DOTA PET and ^18^F-fluorodopa PET may enhance diagnostic capability; however, their availability remains limited in Japan [19-22].

Although international guidelines favor contrast-enhanced CT [9], Japanese guidelines prefer MRI because of safety concerns, as summarized in Table 2 [7,23].

**Table 2:** Comparison between International and Japanese Guidelines.

Category	International guidelines [9]	Japan guidelines [7]
Position of Contrast-Enhanced CT	First-choice imaging diagnostic method.	Generally contraindicated. However, permissible under proper precautions.
Risk Management for Contrast Agent Usage	Preoperative management with alpha-blockers is critical.	Owing to high risks, MRI is recommended.
Role of MRI	Supplementary role (used when CT is unsuitable or under specific conditions).	First-choice imaging diagnostic method.
Background of Guidelines	Contrast-enhanced CT is widely accessible in Western countries, with confidence in safety measures.	MRI is prevalent in Japan, and contrast-enhanced CT is limited to avoid associated risks.

**Figure 4:**
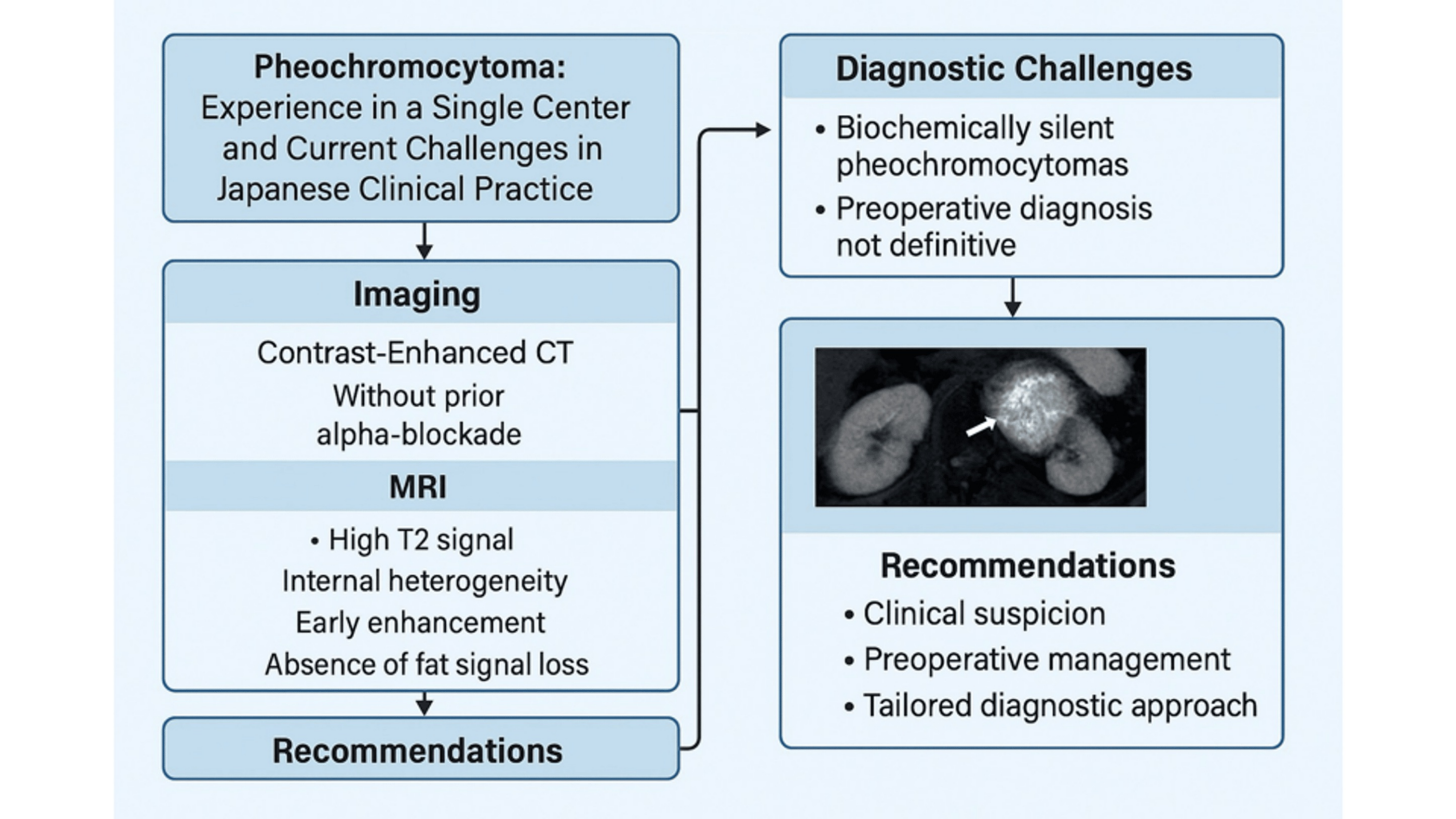
Focus and principle of the study.

An institution-specific, balanced approach is necessary to optimize both diagnostic accuracy and patient safety. The study’s focus and principal findings are summarized in Figure 4.

The study’s limitations included a small sample size, a retrospective, single-center design, and limited data acquisition, all of which may limit generalizability. The effect of alpha-blocker administration on safety has not been quantitatively assessed, and the discussion of advanced imaging techniques has remained limited. These limitations should be considered when interpreting the results. An adrenal tumor should still raise the concern for pheochromocytoma even when MIBG and biochemical evaluation with metanephrines are normal; thus, more careful evaluation of images is required.

## Conclusions

This study identified preoperative diagnostic pitfalls, particularly in small and atypical tumors, and highlighted the value of combining multiple imaging modalities. Accurate diagnosis and management of pheochromocytomas depend on integrating biochemical, imaging, and clinical data. Preoperative alpha blockade is essential, even when catecholamine testing and scintigraphy are inconclusive. Further research is required to evaluate the role and effectiveness of a multimodal imaging approach. These findings may contribute to improving diagnostic accuracy and safety in the clinical management of pheochromocytoma.
